# Cross-Resistance Among Sequential Cancer Therapeutics: An Emerging Issue

**DOI:** 10.3389/fonc.2022.877380

**Published:** 2022-06-23

**Authors:** Rossella Loria, Patrizia Vici, Francesca Sofia Di Lisa, Silvia Soddu, Marcello Maugeri-Saccà, Giulia Bon

**Affiliations:** ^1^ Cellular Network and Molecular Therapeutic Target Unit, IRCCS Regina Elena National Cancer Institute, Rome, Italy; ^2^ Unit of Phase IV Trials, IRCCS Regina Elena National Cancer Institute, Rome, Italy; ^3^ Medical Oncology A, Department of Radiological, Oncological, and Anatomo-Pathological Sciences, Umberto I University Hospital, University Sapienza, Rome, Italy; ^4^ Division of Medical Oncology 2, IRCCS Regina Elena National Cancer Institute, Rome, Italy

**Keywords:** targeted-therapy, cancer therapeutics resistance, cross-resistance, sequential therapeutics, personalized oncology

## Abstract

Over the past two decades, cancer treatment has benefited from having a significant increase in the number of targeted drugs approved by the United States Food and Drug Administration. With the introduction of targeted therapy, a great shift towards a new era has taken place that is characterized by reduced cytotoxicity and improved clinical outcomes compared to traditional chemotherapeutic drugs. At present, targeted therapies and other systemic anti-cancer therapies available (immunotherapy, cytotoxic, endocrine therapies and others) are used alone or in combination in different settings (neoadjuvant, adjuvant, and metastatic). As a result, it is not uncommon for patients affected by an advanced malignancy to receive subsequent anti-cancer therapies. In this challenging complexity of cancer treatment, the clinical pathways of real-life patients are often not as direct as predicted by standard guidelines and clinical trials, and cross-resistance among sequential anti-cancer therapies represents an emerging issue. In this review, we summarize the main cross-resistance events described in the diverse tumor types and provide insight into the molecular mechanisms involved in this process. We also discuss the current challenges and provide perspectives for the research and development of strategies to overcome cross-resistance and proceed towards a personalized approach.

## Introduction

The history of targeted cancer therapy started in the 1970s with the approval of tamoxifen, the first selective estrogen receptor (ER) modulator (1). At the beginning of the ‘80s, advances in molecular biology allowed to identify new molecular targets involved in neoplastic transformation and progression. These discoveries sparked a revolution in cancer therapy, at the time mainly based on combination chemotherapy regimens, that culminated in the development of targeted monoclonal antibodies (mAbs) and selective protein kinase small molecule inhibitors (PKIs) (2). Following the development of hybridoma technology by George Köhler and Cèsar Milstein in 1975 (3), who were awarded a Nobel prize for their discoveries in 1984, several attempts to develop murine mAbs against myelo- and lympho-proliferative diseases and lymphomas did not give the expected results (4, 5). In 1986 the United States (U.S.) Food and Drug Administration (FDA) approved the first therapeutic mAb, muromonab-CD3, which was to be used as an immunosuppressive for prevention of transplant rejection (6). At the beginning of the ‘90s, growing scientific and industrial interest in developing targeted drugs ushered us into an era characterized by the approval of an increasing number of MAbs and PKIs. The first tyrosine-kinase inhibitor (TKI), Imatinib mesylate, directed towards the fusion protein BCR-ABL, obtained approval by the FDA in 2001 (7). Since then, more than 70 PKIs have been introduced (8), and 100 mAbs have been approved by April 2021, with GlaxoSmithKline’s Programmed cell Death protein 1 (PD1) blocker dostarlimab (9). More recently, checkpoint inhibitory mAbs and chimeric antigen-specific receptor (CAR)-transfected T-cells (CAR-T cells) have also had impact in the oncology field (10).

Targeted cancer therapy has provided huge benefits in terms of improved response and survival rates as well as reduced side effects compared to traditional chemotherapy. However, one of the greatest drawbacks to all currently available cancer therapies is the emergence of drug resistance leading to tumor progression (11). For this reason, many patients affected by advanced malignancy receive sequential anti-cancer therapies, which may include chemotherapy, immunotherapy, targeted therapy, endocrine therapy, or a combination of them. The complexity requires strict criteria to define and enumerate the sequential lines of therapy uniformly across solid malignancies (12). From a mechanistic point of view, recent high-throughput sequencing studies and quantitative modeling approaches have revealed extensive intratumor heterogeneity and highly dynamic tumor clonal evolution under the selective pressure exerted by drug treatments (13–15). It is therefore easy to anticipate that the evolutionary trajectories imposed by drugs may intersect through subsequent lines of treatment in unpredictable ways. In this scenario, the probability that cross-resistance emerges between sequential treatments increases with a higher number of therapeutic possibilities. Unfortunately, the current adoption of sequential lines of therapy according to guidelines is a strategy that does not consider cross-resistance as well as the possible development of new targetable vulnerabilities (16).

In this review, we summarize the main known events of cross-resistance and the molecular mechanisms involved. We also provide an overview of real-world data (RWD) as a tool to address the complexity of cancer therapy, and the possible strategies to adopt in an attempt to overcome or prevent cross-resistance.

## Cross-Resistance Among Cancer Therapeutics

**Figure 1:**
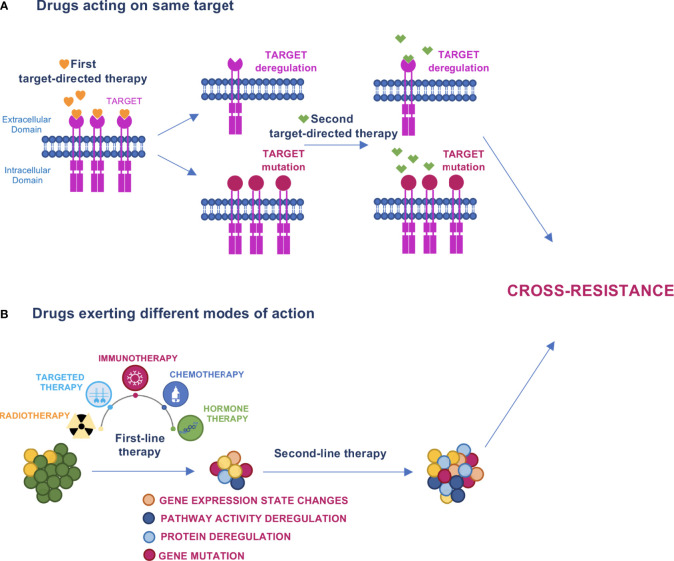
Models of cross–resistance. **(A)** When drugs acting on the same target are sequentially administered, the first drug can induce target deregulation or mutation that causes escape from therapy and cross–resistance to the subsequent drug. **(B)** Cancer therapy promotes evolutionary dynamics fostering mutations, protein or pathway activity deregulations, and changes in gene expression states that can determine cross–resistance to the next treatment.

Cross-resistance occurs when acquired resistance induced by a drug treatment results in resistance to other drugs (**Figure 1**). It may occur in the sequential administration of agents with overlapping working mechanisms, such as receptor tyrosine kinase erbB-2 (HER2)-targeting agents trastuzumab+pertuzumab and trastuzumab-emtansine (T-DM1) in breast cancer (BC). In this case, T-DM1 second line treatment might have reduced efficacy. In a more complex scenario, the characterization of tumor evolution in terms of clonal selection during therapy has revealed that under prolonged drug exposure, cancer cells enter a drug-tolerant state known as drug tolerant persister cells (DTPCs) (17). At this stage, the activation of heterogeneous mechanisms of drug resistance causes these subclones to expand and generate stable resistant cell populations (17–19). The sensitivity of these populations to subsequent drugs is difficult to predict unless biomarkers will be defined to represent specific collateral trajectories. The main events of cross-resistance described thus far for the different types of targeted therapies are reported below.

### Chemotherapeutic Drugs

The use of cytotoxic/cytostatic chemotherapy was the first approach adopted in the treatment of tumors. However, the effectiveness of these drugs was often limited by the emergence of multiple drug resistance (MDR) (20) which determined cross-resistance to diverse structurally and functionally unrelated chemotherapeutic agents.

Although cancer cells develop various mechanisms to escape chemotherapy, drug transporters belonging to ATP-binding cassette (ABC) family are the main players implicated in MDR. These ATP-dependent efflux pumps actively remove drugs from cancer cells (21). Glycoprotein P (P-gp) is the most relevant ABC drug transporter. It is encoded by the *multidrug resistance protein 1* gene (*MDR1, ABCB1*) and overexpressed in over 50% of cancers with a MDR phenotype (22). P-gp overexpression has been implicated in resistance to approximately 20 different cytotoxic drugs including doxorubicin, paclitaxel and related taxane drugs (23). Many anticancer drugs have been reported to induce the up-regulation of Forkhead Box O3 (FOXO3A), a transcription factor closely implicated in MDR, that in turn enhances *ABCB1* transcription and P-gp expression (24).

Other ABC family members involved in MDR include Breast Cancer Resistance Protein (BCRP; also known as mitoxantrone resistance protein, MXR), and multidrug resistance-associated proteins (MRPs) (25). BCRP (encoded by the *ABCG2* gene) is the second most relevant drug transporter. Its overexpression has been described in many cancers including breast and ovarian and is associated with resistance to mitoxantrone and topotecan (26, 27). MRPs include MRP1 and MRP2 (also known as MDR-related protein 1 and MDR-related protein 2) encoded respectively by the *ABCC1* and *ABCC2* genes (21, 25, 28). The drug resistance spectra of MRP1 is similar to that of P-gp except for taxanes, while MRP2 confers resistance to MRP1 substrates and cisplatin, one of the most frequently used drugs in cancer therapy (23, 26).

DNA damage repair (DDR) genes have been implicated in the cross-resistance among chemotherapeutic drugs. In multiple mouse models of NSCLC, prolonged cisplatin treatment promoted the emergence of resistant tumors that were cross-resistant to platinum analogs. These cisplatin-resistant tumors showed enhanced DNA repair capacity due to elevated levels of multiple DDR-related genes (29). In support of these findings, the DNA repair capacity measured in peripheral lymphocytes is an independent predictor of survival for non-small cell lung cancer (NSCLC) patients treated with platinum-based chemotherapy (30) and the inhibition of DNA repair kinases could also prevent doxorubicin resistance in BC cells (31). Furthermore, DDR pathways can be enhanced in cancer cells providing a survival advantage after chemotherapy (32).

### HER2- and Estrogen Receptor-Targeted Therapies

HER2 is a member of the Epidermal Growth Factor Receptor (EGFR) family of receptor tyrosine kinases. HER2 amplification and/or overexpression have been described in BC (20% of cases) and in a variety of other solid tumors, including gastric cancer (GC, 20%), biliary tract cancer (BTC, 20%), bladder cancer (BlC, 12.5%), colorectal cancer (CRC, 5%) and NSCLC (2.5%) (33). Although HER2 is an established therapeutic target in a subset of women with BC, the early HER2-targeted therapies have not proven to be as effective in HER2-positive (HER2+) GC or other solid tumors.

**Figure 2:**
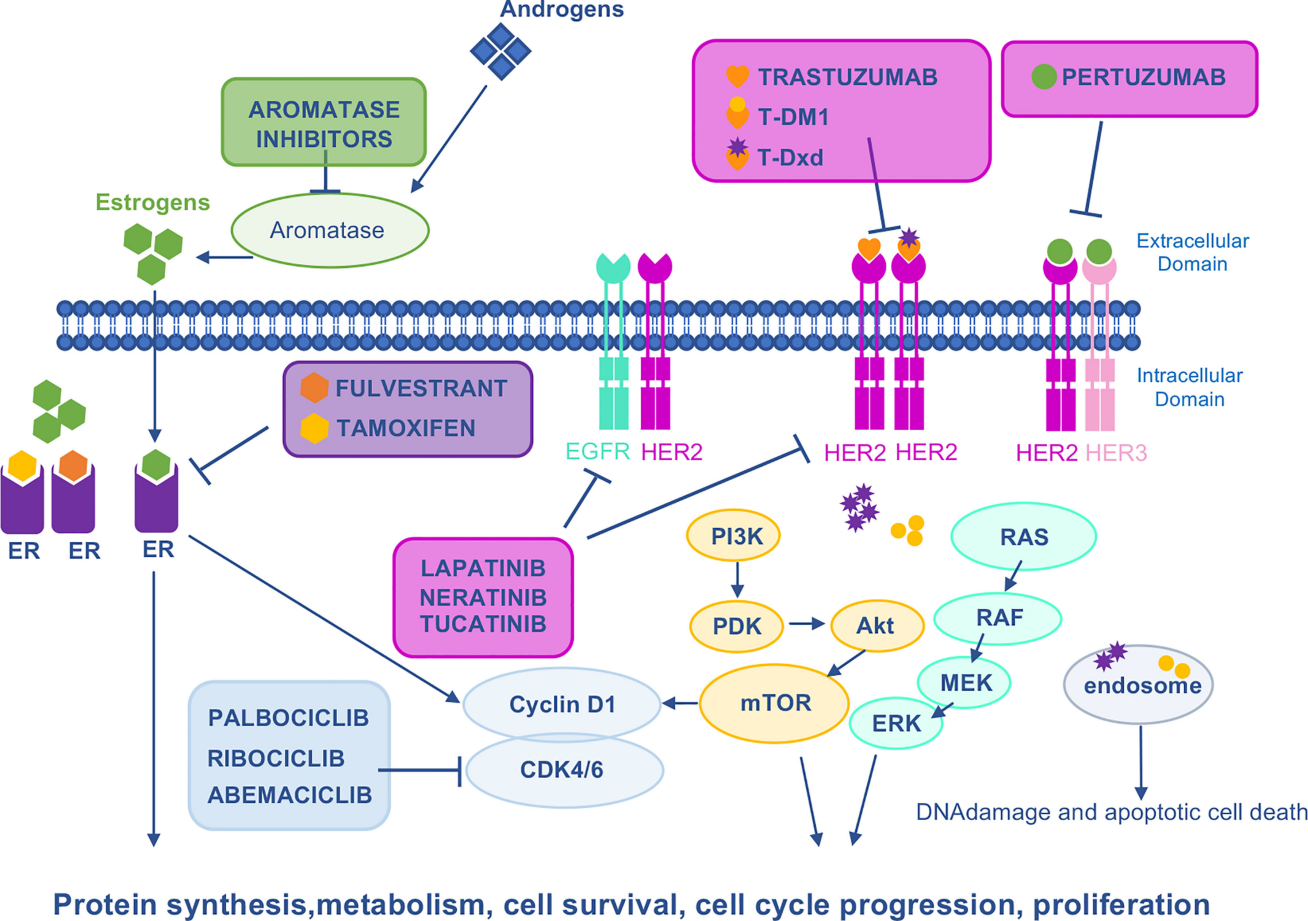
Mechanism of action of HER2–, ER–, and CDK4/6–targeted drugs. HER family RTKs (EGFR, HER2, and HER3) activate several oncogenic signaling pathways such as Ras/Raf/MEK/ERK or PI3K/Akt/mTOR to stimulate growth and proliferation. Direct HER2 inhibitors include trastuzumab and the conjugates of trastuzumab with DM1 (T–DM1) or Dxd (T–Dxd). In the case of drug–antibody conjugates, upon binding of trastuzumab to HER2, the payload is internalized by endocytosis to induce DNA damage. Pertuzumab mAb binds HER2, preventing homodimerization and heterodimerization with other family members, especially HER3. Lapatinib is a EGFR/HER2 TKI that attenuates cell proliferation, cell–cycle regulation, and downstream pathways. Tucatinib is a selective HER2 TKI with minimal inhibition of EGFR. Neratinib is a pan–HER irreversible TKI. ER is a transcription factor which, under estrogen stimulation, is recruited on the promoter of its target genes to induce cell proliferation. Aromatase inhibitors prevent the aromatase–dependent conversion of androgens to estrogens, whereas fulvestrant and tamoxifen are both anti–estrogens that counteract the effects of estrogen by directly binding to the ER. CDK4 and CDK6 form complexes with CyclinD1 to stimulate proliferation. Palbociclib, ribociclib, and abemaciclib are CDK4/6 small molecule inhibitors. CDK 4/6, cyclin–dependent kinases 4/6; DM1, derivative of maytansine 1; Dxd, deruxtecan; EGFR, epidermal growth factor receptor; ER, estrogen receptor; ERK, extracellular–signal regulated kinase; HER2, human epidermal growth factor 2; HER3, human epidermal growth factor 3; MEK, mitogen–activated protein kinase kinase; mTOR, mammalian target of rapamycin; PDK, phosphatidylinositol–dependent kinase; PI3K, phosphoinositide 3–kinase; RAF, rapidly accelerated fibrosarcoma; RAS, RAS proto–oncogene.

Since the approval of trastuzumab, the first anti-HER2 agent (34) for BC treatment in 1998, an array of other anti-HER2 agents, such as pertuzumab, lapatinib, T-DM1, and trastuzumab- Deruxtecan (T-Dxd) mAb-drug conjugates (ADCs) and others have been approved, significantly improving the outcome of BC patients (**Figure 2**). In addition, a widening arsenal of novel HER2-targeting drugs are under development (35). Anti-HER2 treatments are administered in neoadjuvant, adjuvant, and advanced settings of BC patients. However, there is a growing body of evidence suggesting that HER2-targeted treatment may significantly influence the loss/reduction of HER2 expression (36–47). Mittendorf and colleagues have described the loss of HER2 amplification in residual disease in 32% of BC patients treated with neoadjuvant trastuzumab in combination with anthracyclines and taxanes, as this change is associated with poor recurrence-free survival (43). In a retrospective cohort study involving 21,755 Japanese BC patients, loss of HER2 was observed in 20.4% following neoadjuvant trastuzumab (44). In the advanced setting, Ignatov and colleagues have shown that loss of HER2 is associated with previous HER2-targeted treatment and reduced disease-free survival. Interestingly, a change in HER2 expression was observed in 47.3% of trastuzumab-treated patients and in 63.2% of trastuzumab plus pertuzumab-treated ones (46). In concordance, reduced T-DM1 efficacy has been described in HER2+ advanced BC patients previously treated with dual HER2 blockade by trastuzumab plus pertuzumab combination as compared to trastuzumab alone (47–51). At the molecular level, a marked reduction of HER2 expression on cell membrane and HER2 nuclear translocation have been shown to account for cross-resistance between trastuzumab plus pertuzumab and T-DM1 (47). In agreement with reduced expression of HER2 on trastuzumab plus pertuzumab rather than its loss, T-Dxd showed a remarkable improvement in progression-free survival (PFS) vs T-DM1 in second-line treatment for previously treated BC patients (preliminary results from DESTINY Breast 03 trial, The Asco Post, posted 9/19/21). This striking result is probably due to the unique linker-payload system of T-Dxd, that contributes to its preclinical efficacy against tumors with low HER2 expression (52).

Another possible explanation for cross-resistance among subsequent HER2-targeted drugs is represented by the clonal evolution under the selective pressure of treatments. In this case, based on tumor heterogeneity, trastuzumab or other HER2-targeting drugs preferentially eradicate HER2+ clonal populations selecting the HER2-negative ones, that in turn emerge and drive tumor progression (41, 42, 44).

Similar cross-resistance has been reported between other HER2-targeting agents. Neratinib is an irreversible HER2 TKI approved for adjuvant treatment of HER2+/estrogen receptor-positive (ER+) early BC following adjuvant-trastuzumab-based therapy, and, in combination with capecitabine, for HER2+ metastatic BC patients who have received two or more prior anti-HER2-based regimens in the metastatic setting. Evidence from a pre-clinical model of neratinib-resistant BC cell lines indicates cross-resistance to trastuzumab and lapatinib. This cross-resistance is bi-directional, as lapatinib- and trastuzumab-resistant cells are also cross-resistant to neratinib (53). In agreement, in phase II studies, drug-naïve patients responded better to neratinib than patients previously treated with trastuzumab (54) or with lapatinib (55).

Although the incidence of HER2+ disease in patients with GC is similar to that observed in patients with BC, the success rate achieved in BC with several HER2-targeted therapies has not yet been observed in GC. This might be explained by biological differences among these tumor types, such as the pattern of expression of HER2, or the higher degree of intratumoral heterogeneity of HER2 expression in GC compared to BC (56). Nevertheless, based on data from the ToGA trial, the combination of chemotherapy plus trastuzumab represents the standard of care for first-line treatment of HER2+ advanced GC (57). By contrast, HER2-targeted ADCs explored in the second-line setting showed promising results; in January 2021, based on the robust data from DESTINY-Gastric01 phase II trial, the U.S. FDA approved T-Dxd ADC for patients with metastatic GC who have received a prior trastuzumab-based regimen (58). Although the introduction of T-Dxd has represented an important step forward, the benefit in this setting was much higher in patients with a HER2 score of 3+ on immunohistochemical analysis (IHC), while a lower response rate was observed in patients with a 2+ score with positive results on *in situ* hybridization (58% vs 29%) (58, 59). It is worth noting that in this study the HER2 status was evaluated using archival tissue specimens and thus the HER2 status immediately prior to T-Dxd administration had not been investigated. Indeed, similarly to what has been described in BC, loss of HER2 expression after trastuzumab treatment has been reported in patients with HER2+ advanced GC (60–63).

Another mechanism of cross-resistance among HER-2 targeted therapies involves the emergence of the *HER2 L755S* variant after therapy. L755S is an activating mutation of *HER2* accounting for 60% of *HER2* mutations found in metastatic BCs (64). Recent studies have described the emergence of *HER2 L755S* under the pressure of lapatinib and trastuzumab that results in cross-resistance to other single agents or combination HER2-targeted therapy, both in the pre-clinical and patient-derived models (65, 66). Similarly, no significant response to trastuzumab has been observed in HER2+ metastatic BC patients whose tumors harbor *HER2* mutations (67). In supporting the association of *HER2* mutations with trastuzumab resistance, the frequency of acquired *HER2* mutations in patients with advanced BC after trastuzumab treatment is much higher compared to patients with early-stage tumors, and an enrichment of *HER2* mutations in metastatic lesions from patients undergoing adjuvant trastuzumab has been reported (64, 68).

*HER2* mutations account for cross-resistance also in HER2 non-amplified BC patients. In BC patients, about 70% of *HER2* mutations have been found in metastatic ER+/HER2 non-amplified tumors, suggesting that the emergence of *HER2* mutations may represent a mechanism of acquired resistance to endocrine therapy (69). In line with this, Nayar and colleagues described the appearance of *HER2* mutations in metastatic lesions from eight ER+ BC patients under the selective pressure of ER-directed aromatase inhibitors, tamoxifen, or fulvestrant. An *in vitro* analysis showed that *HER2* mutations confer estrogen independence and resistance to tamoxifen, fulvestrant, and to the Cycline Dependent Kinase 4 (CDK4)/Cycline Dependent Kinase 6 (CDK6) inhibitor palbociclib, which was overcome by combining ER-therapy with the HER2-inhibitor neratinib (70). Overall, these data indicate that acquired *HER2* mutations account for cross resistance in *i*) HER2+ BC patients treated with HER2-targeting agents, where they are potentially useful biomarkers of trastuzumab/lapatinib resistance in subsequent lines of treatments; *ii*) HER2- BC patients treated with endocrine therapy.

**Table 1** summarizes the cross-resistance events described between sequential HER2-targeted therapies and ER-targeted therapies and between ER-targeted agents and the CDK4/CDK6 inhibitor Palbociclib.

**Table 1:** Cross–resistance in HER2– and ER–targeted therapies.

Previous agent	Subsequent agent	Ref.	Type of study	Proposed mechanism	Supporting literature
Trastuzumab+pertuzumab	T–DM1	*Bon G*, *J Exp Clin Cancer Res 2020* (47)	*In vitro* BT474 and SkBr3 BC resistant cell model + Observational	Reduction of membrane HER2 expression; HER2 nuclear translocation	*Burstein HJ, J Clin Oncol 2003* (36) *Pectasides D, Anticancer Res 2006* (37) *Hurley J, J Clin Oncol 2006* (38) *Harris LN, Clin Cancer Res 2007* (39) *van de Ven S, Cancer Treat Rev 2011* (40) *Niikura N, Ann Oncol 2016* (41) *Mittendorf EA, Clin Cancer Res 2009* (43) *Gahlaut R, Eur J Cancer 2016* (45) *Ignatov T, Breast Cancer Res and Treat 2019* (46) *Pietrantonio F, Int J Cancer 2016* (60) *Saeki H, Eur J Cancer 2018* (61) *Seo S, Gastric Cancer 2019* (62) *Kijima T, Anticancer Res 2020* (63)
		*Vici*, *Oncotarget 2017* (48)	Observational		
		*Noda–Narita S, Breast Cancer 2019* (50)	Observational		
		*Pizzuti L*, *Ther Adv Med Oncol 2021* (51)	Observational		
Neratinib	Trastuzumab Lapatinib	*Breslin S*, *British J Cancer 2017* (53)	*In vitro* cell model	Increased cytochrome CYP3A4 activity *bidirectional*	*Burstein HJ, J Clin Oncol 2010* (54) *Awada A, Ann Oncol* (55)
Lapatinib	Trastuzumab	*Cocco E*, *Sci Signal 2018* (66)	*In vitro* BT474 and SkBr3 BC resistant cell model + patient analysis	Emergence of HER2 L755S mutation *bidirectional*	*Zuo WJ, Clin. Cancer Res 2016* (64) *Xu X, Clin Cancer Res 2017* (65) *Boulbes DR, Mol Oncol 2015* (67) *Yi Z, Breast Cancer 2020* (68)
Aromatase inhibitors Tamoxifen Fulvestrant	Tamoxifen Fulvestrant Palcociclib	*Nayar U*, *Nat genetics 2019* (70)	*In vitro HER2*–mutated T47D and MCF7 BC cell model + patient analysis	Emergence of HER2 L755S, V777L, L869A, and S653C mutation	*Croessmann S, Clin Cancer Res 2019* (69)

### CD4/6 Inhibitors

The clinical management of ER+ BC (mainly Luminal A and Luminal B) includes endocrine therapy (ER downregulators, selective ER modulators, and aromatase inhibitors) as primary treatment, albeit luminal B tumors are mainly treated with chemotherapy due to lower sensitivity to endocrine therapy (71). However, resistance to endocrine therapy has been shown to be dependent on the Cyclin D-CDK4/6 pathway (72). On this basis, three CDK4/6 inhibitors, namely palbociclib (73), ribociclib (74), and abemaciclib (75) have been FDA approved in combination with endocrine therapy for the first- or second-line treatment of ER+ HER2- advanced BC (**Figure 2**). In an *in vitro* model of ER+ HER2- BC cell lines, cross-resistance among different CDKis has been reported, but not between CDK inhibition and chemotherapeutic agents (76) (**Table 2**). Loss or dysregulation of Retinoblastoma-associated Protein 1 (RB1) have been demonstrated to emerge under selective pressure from CDK4/6 inhibitors potentially conferring therapeutic resistance (77, 78). Whether continuing a CDK4/6 inhibitor beyond progression may prove to be an effective strategy is currently being tested by several ongoing phase I and II trials (MAINTAIN NCT02632045, PACE NCT03147287, NCT01857193, NCT 02871791, and TRINITI-1 NCT 02732119).

**Table 2:** Cross–resistance in CDK4/6–targeted therapies.

Previous agent	Subsequent agent	Ref.	Type of study	Proposed mechanism	Supporting literature
Palbociclib	Abemaciclib	Ogata R, *Breast Cancer* 2021 (70)	*In vitro* MCF7 and KPL4 BC resistant cell model	Downregulated retinoblastoma protein RB. Hypothethical	*Condorelli R, Ann Oncol. 2018* (71) *Pandey K, Int J Cancer 2019* (78)
Ribociclib	Alpelisib	*Costa C*, *Cancer Discov 2020* (72)	Patient analysis + CRISPR PTEN KO T47D and MCF7 BC cell and mouse model	Loss of PTEN, that results in p27 exclusion from the nucleus and increased activation of CDK2 and CDK4	Razavi P, *Nat Cancer* 2020 (73) Juric D, Nature 2015 (74)

Recently, clinical cross-resistance mediated by PTEN loss has been shown between CDK4/6 inhibitors and alpelisib, an alpha-specific PI3K inhibitor (PI3Ki) recently approved for the treatment of PIK3CA-mutated ER+ advanced BC that progressed on previous endocrine therapy (79, 80) (**Table 2**). Costa and colleagues demonstrated that loss of Phosphatase and Tensin Homolog (PTEN) promotes translocation of p27 outside the nucleus by raising AKT activity, which in turn increases CDK4/6 activity, ultimately overcoming the blockade of CDK4/6. PTEN loss had been shown to cause resistance to PhosphatidylInositol 3-Kinase (PI3K) inhibition in previous studies (81, 82).

### EGFR-Targeted Therapies

EGFR overexpression has been reported in diverse tumor types including head and neck, ovarian and cervical cancers, Bladder Cancer and CRC, where it has been associated with poor outcomes and prognosis (83). Furthermore, driver *EGFR* activating mutations are common in NSCLC (84) and occur in 3% of CRC (85). For these reasons EGFR became a popular therapeutic target; both EGFR-targeted mAbs and TKIs demonstrated efficacy in large phase III clinical trials and were approved for treating lung, colorectal and head and neck cancers.

**Figure 3:**
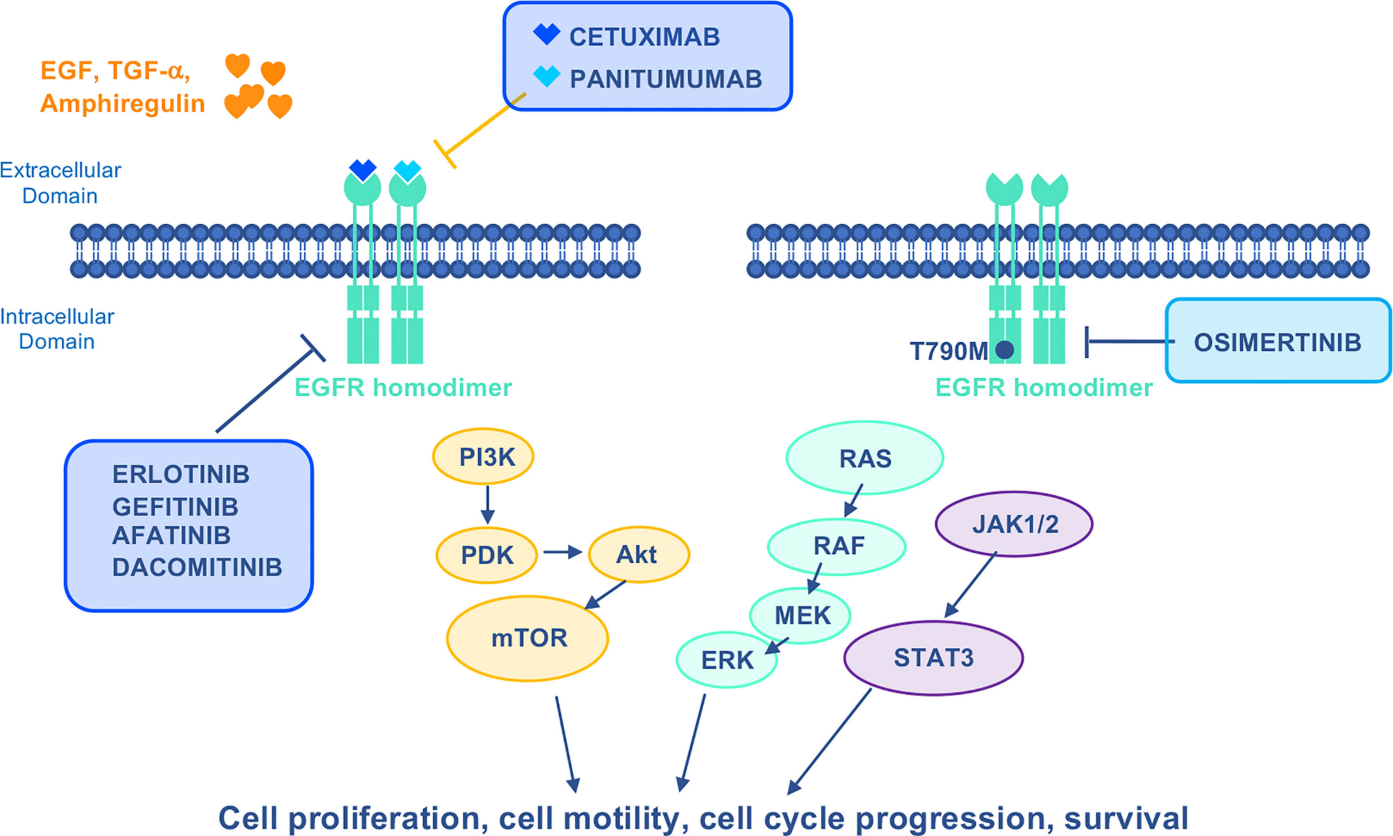
Mechanism of action of EGFR–targeted drugs. EGFR activates Ras/Raf/MEK/ERK, PI3K/Akt/mTOR, and JACK1/2/STAT3 oncogenic signaling pathways to stimulate growth and proliferation. Cetuximab and Panitumumab are mAbs that specifically inhibit EGFR. First–generation reversible (gefitinib and erlotinib) and second–generation irreversible (afatinib and dacomitinib) TKIs were developed to target mutant EGFR. The third generation irreversible TKI Osimertinib is highly selective for EGFR– activating mutations as well as the EGFR T790M mutation. EGF, epidermal growth factor; JACK1/2, janus kinases 1/2; STAT3, signal transducer and activator of transcription 3; TGF–α, transforming growth factor alpha.

EGFR-specific first-generation (gefitinib and erlotinib) or second-generation (afatinib and dacomitinib) TKIs were developed for treatment of patients with metastatic, *EGFR*-mutated NSCLC (86). Given that up to 60% of patients progressing on TKIs acquire the secondary *EGFR T790M* mutation (87), the third generation irreversible EGFR TKI osimertinib was developed which demonstrated clinical activity in *T790M* patients who had progressed on previous TKIs (**Figure 3**). Recently, based on results from the FLAURA trial showing OS benefit over first-generation TKIs, upfront use of osimertinib became the standard of care (88).

Concomitantly with the introduction of osimertinib in the clinical practice, cross-resistance has been reported between gefitinib and irreversible EGFR-TKIs in human lung cancer cells (89). (**Table 3**) Mechanistically, in a gefitinib-resistant cell model, *Kelch Like ECH Associated Protein 1* (*KEAP1)* gene mutation disrupts the KEAP1-Nuclear factor erythroid 2-Related Factor 2 (NRF2) oncogenic signaling pathway leading to constitutive activation of NRF2, cell proliferation, and resistance to gefitinib as well as cross-resistance to afatinib and osimertinib. Somatic mutations in the *NFE2L2* (encoding NRF2) and *KEAP1* genes have been described in 23% of patients with lung adenocarcinoma (LAC) (84) and are usually mutually exclusive. Mutations in the KEAP1-NRF2 pathway have been associated with worse clinical outcomes and earlier disease progression to chemotherapy in LAC patients (90). More importantly, the emergence of KEAP1 loss/NRF2 activation has been reported as a mechanism of acquired resistance to EGFR-TKIs in *EGFR*-mutated LAC cells (91, 92) and patients (93). Furthermore, patients with *KEAP1-NFE2L2*-mutant tumors have shorter recurrence-free interval on treatment with EGFR TKI (94) and KEAP1 inactivation reduces the sensitivity of EGFR-driven tumors to osimertinib in an EGFR-driven Trp53-deficient LAC mouse model (95). Overall, these results suggest the involvement of *KEAP1-NFE2L2* genetic alterations in cross-resistance occurring between first-generation and third-generation irreversible EGFR TKIs, that has been shown to be overcome with the introduction of osimertinib as first-line treatment. The post-osimertinib treatment options for EGFR-mutated NSCLC including innovative drugs or combination therapies are under investigation in ongoing clinical trials (96).

**Table 3:** Cross–resistance in EGFR–targeted therapies.

Previous agent	Subsequent agent	Ref.	Type of study	Proposed mechanism	Supporting literature
Gefitinib	Afatinib Osimertinib	*Park SH, FASEB J. 2018* (89)	*In vitro* HCC827 NSCLC resistant cell model + *In vivo* resistant–NSCLC mouse model	*KEAP1* mutation leading to constitutive activation of NRF2	*Krall EB, Elife 2017* (91) *Yamadori T, Oncogene 2012* (92) *Yu HA, Clin Cancer Res 2018* (93) *Hellyer JA, Lung Cancer 2019* (94) *Foggetti G, Cancer Discov 2021* (95)
Cetuximab	Panitumumab	*Arena S, Clin Cancer Res. 2015* (98)	*In vitro* CRC resistant cell model and *EGFR*–mutated CRC cell model + patient analysis	Emergence of *EGFR* S464L, G465R, and I491M mutations	*Van Emburgh BO, Nat Commun 2016* (99) *Misale S, Cancer Discov 2014* (100) *Montagut C, Nature Med 2012* (101)
Cetuximab Panitumumab	Cetuximab Panitumumab	*Diaz LA Jr, Nature 2012* (102)	Patients’ sera analysis	Emergence of *KRAS* mutations, indirect evidence. Presumably bidirectional	*Peeters M, Eur J Cancer 2015* (105) *De Roock W, Lancet Oncol 2015* (106)
		*Misale S, Nature 2012* (103)	*In vitro* DiFi and Lim1215 CRC resistant cell model+ patient analysis		
		*Van Cutsem E, J Clin Oncol* 2011 (104)	Phase III clinical trial		

Cetuximab and panitumumab EGFR-targeted mAbs have been approved in combination with chemotherapy for the first-line treatment of Kirsten RAt Sarcoma (KRAS) wt CRC (**Figure 3**). They can also be administered as monotherapy upon progression following prior chemotherapeutic regimens. Despite clinical benefits obtained in CRC by combining EGFR-targeted mAbs and chemotherapy, this has been shown to last 8-10 months due to drug resistance (97). Multiple *EGFR* and *RAS* mutations were among the mechanisms of resistance reported (98, 99). *EGFR* acquired mutations preferentially occur in the extracellular domain, which impair antibody-binding (100). Among the different specific mutations identified in cetuximab-resistant CRC patients, some proved to be permissive for panitumumab binding, whereas others determined cross-resistance (98, 101). The emergence of *RAS* mutations induced by anti-EGFR therapies has been reported in approximately 50% of patients with RASwt CRC and is responsible for acquired resistance to cetuximab (102, 103) (**Table 3**). *RAS* mutations can result in constitutive activation of RAS-associated signaling that renders anti-EGFR therapies ineffective for CRC. Consistent with this, the predictive role of *RAS* mutations in the clinical responses of CRC to anti-EGFR therapies has been demonstrated in several pivotal studies (104, 106).

### Androgen Receptor-Targeted Therapies

**Figure 4:**
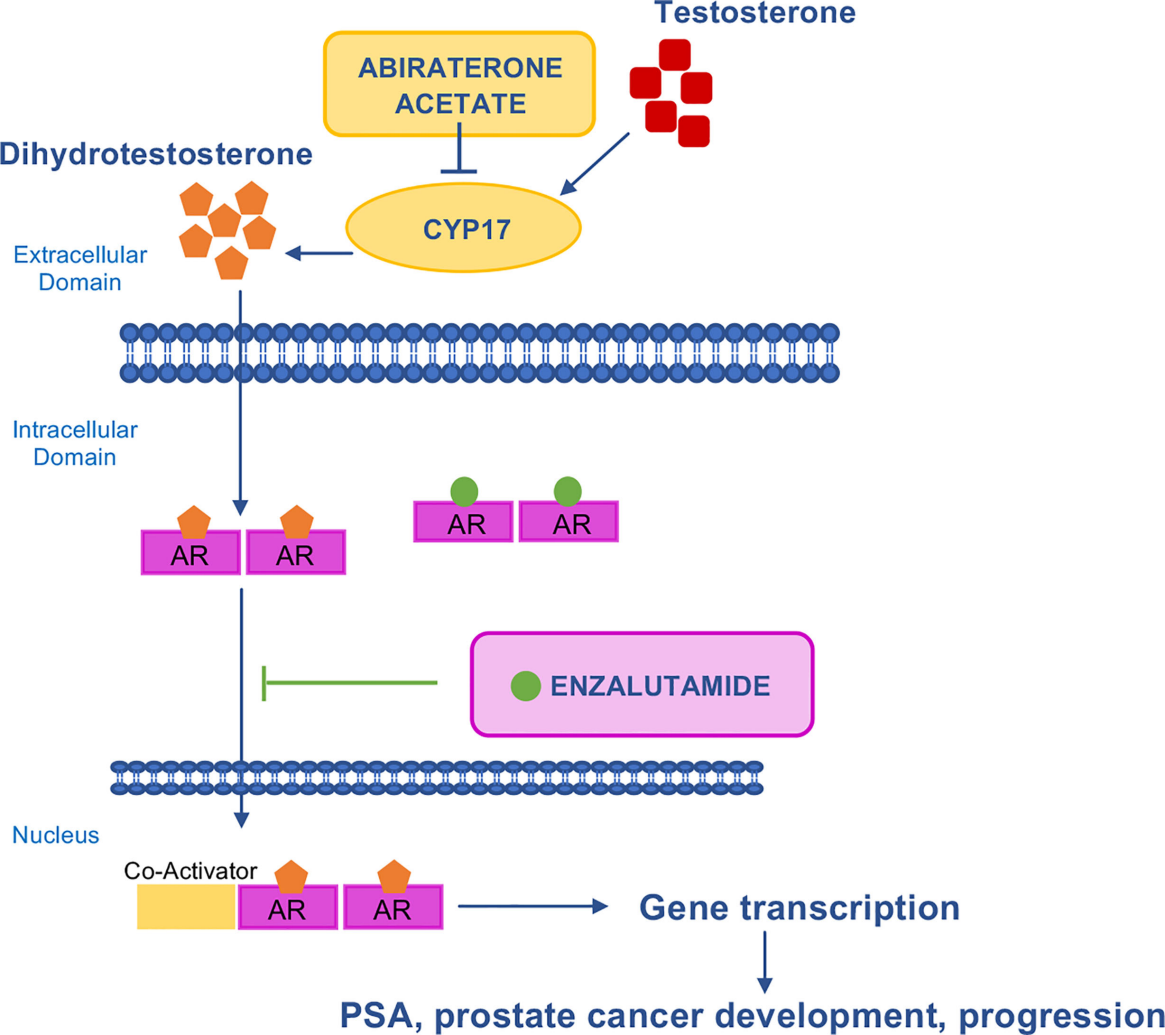
Mechanism of action of AR–targeted drugs. AR is a transcription factor that is activated by androgenic hormones binding. Upon activation, AR translocates into the nucleus where it activates the transcription of genes involved in cancer development and progression. Abiraterone acetate inhibits CYP17, the enzyme responsible for the conversion of testosterone to dihydrotestosterone. Enzalutamide is a potent, competitive binder of androgens at the AR. It prevents the translocation of the AR from the cytoplasm to the nucleus. AR, androgen receptor; CYP17, 17 α–hydroxilase/C17,20–lyase; PSA, prostate–specific antigen.

Prostate cancer (PC) is the most common cancer in men and is dependent on the Androgen Receptor (AR) signaling for its growth and progression (107). For this reason, androgen deprivation represents the gold standard first-line treatment for PC patients. Progression is due to transition from a hormone sensitive stage to castration resistant disease (CRPC) (108). Over the past decade, multiple treatment options have demonstrated clinical efficacy in metastatic hormone sensitive PC (mHSPC), non-metastatic CRPC (nmCRPC) and metastatic CRPC (mCRPC) (109). The development of novel, highly potent AR signaling inhibitors (ARSIs) such as enzalutamide and abiraterone acetate (FDA approved in 2012, and 2018 respectively) (**Figure 4**) has represented a major step towards more efficient inhibition of AR signaling and conferred survival benefit in mCRPC and nmCRPC patients (110). Taxanes represent the other class of current treatments for CRPC.

More recently, ARSIs have also been approved in hormone-sensitive disease (111–113). With the adoption of ARSIs in early disease, cross-resistance to sequential ARSI treatment has rapidly emerged as a limitation in the sequential use of AR-targeted therapies (110), however the optimal sequence of available ARSIs and taxane-based chemotherapy have not yet been defined (114). Data from pre-clinical models of abiraterone acetate- and enzalutamide-resistant CRPC confirmed cross-resistance among ARSIs (115, 116) and showed cross-resistance between ARSIs and docetaxel but not carbazitaxel (117, 118) (**Table 4**).

**Table 4:** Cross–resistance in AR–targeted therapies.

Previous agent	Subsequent agent	Ref.	Type of study	Proposed mechanism	Supporting literature
Enzalutamide	Abiraterone	*Lombard AP*, *Mol Cancer Ther 2018* (115)	*In vitro* CRPC resistant cell model	Emergence of constitutively active AR variants Bidirectional	*Liu C, Mol Cancer Ther 2019* (119) *Antonarakis ES, J Clin Oncol 2017* (121) *Guo Z, Cancer Res 2009* (123) *Azad AA, Clin Cancer Res 2015* (124) *Joseph JD, Cancer Discov 2013* (125) *Antonarakis ES, N Engl J Med 2014* (129)
Enzalutamide Abiraterone	Apalutamide Darolutamide	*Zhao J*, *Mol Cancer Ther 2020* (116)	*In vitro* CRPC resistant cell model	Activation of the axis AKR1C3/AR–V7 constitutively active variant	
Enzalutamide	Docetaxel	*van Soest RJ*, *Eur J Cancer 2013* (117)	*In vitro* PC346C resistant and HEP3B PC cell model	Overlapping mechanism of action (inhibition of AR nuclear translocation)	*Mezynski J, Ann Oncol 2012* (126) *Schweizer MT, Eur Urol 2014* (127) *van Soest RJ Eur. Urol 2015* (128)

Mechanistically, cross-resistance among enzalutamide and abiraterone acetate is mainly caused by the re-activation of AR pathway by the emergence of AR constitutively active splice variants. Zhao and colleagues demonstrated the involvement of the AR splice-variant 7 (AR-V7) and identified a Aldo-Keto Reductase family 1 member C3 (AKR1C3)/AR-V7 axis, in which AKR1C3 plays a dual function: first, it catalyzes androgen synthesis; second, it binds AR-V7 promoting its stabilization (116, 119). These data indicate that the AKR1C3/AR-V7 axis plays critical roles in cross-resistance between enzalutamide and abiraterone acetate. In addition, patients treated with enzalutamide or abiraterone acetate showed inferior OS and PFS if they were AR-V7 positive rather than AR-V7 negative (120, 121). On the other hand, AR splice variants do not affect sensitivity to chemotherapy: similar overall survival (OS) and PFS were observed in AR-V7 positive and negative patients receiving taxanes (122). Accordingly, AR alterations including gene aberrations and constitutively active splice variants arising from prolonged ARSIs treatment have been widely implicated in the development of resistance to ARSIs (110, 116, 120, 123–125).

The efficacy of chemotherapy after ARSIs treatment has been investigated in multiple retrospective studies. Overall, clinical evidence showed reduced efficacy of docetaxel in CRPC patients previously treated with enzalutamide or abiraterone acetate (126, 127). Mechanistically, inhibition of AR nuclear translocation may be implicated in cross-resistance as a common mechanism of action of AR-targeting agents and docetaxel (117). Conversely, cabazitaxel efficacy is not affected by prior ARSIs treatment (128).

Moreover, based on clinical evidence, it is widely recognized that enzalutamide administration after abiraterone acetate is of greater clinical benefit than *vice versa* (129, 130), whereas the CARD trial showed that switching to taxane chemotherapy is preferred after ARSI failure (130).

### MAPK Inhibitors

**Figure 5:**
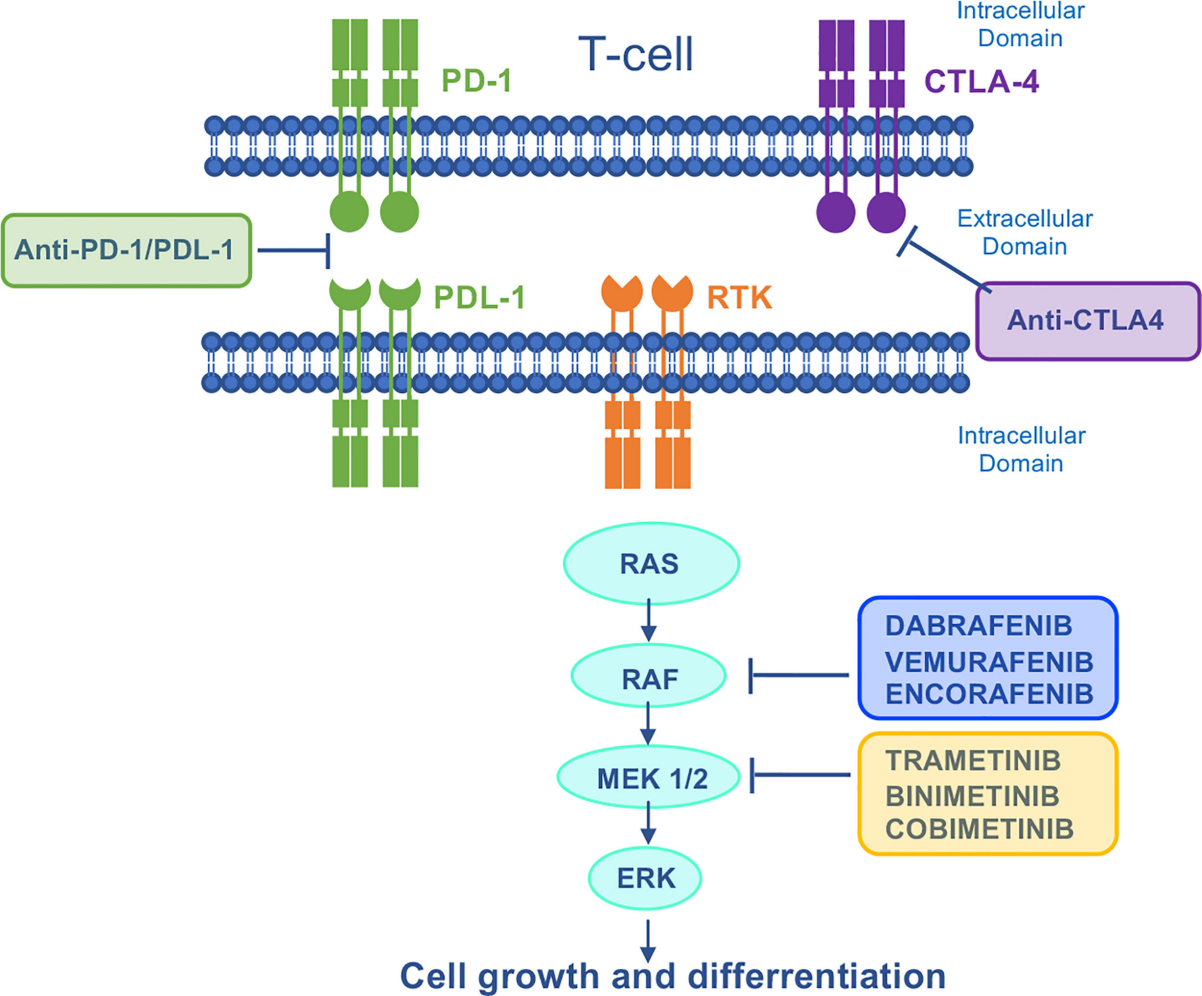
Mechanism of action of MAPK–targeted drugs and immunotherapies. The Ras/Raf/MEK/ERK signaling pathway is activated by several upstream receptor tyrosine kinases. Dabrafenib, vemurafenib and encorafenib are specific BRAF–inhibitors used in the treatment of BRAF–mutant melanoma. In a strategy to vertically target the MAPK signaling pathway, they are used in combination with trametinib, cobimetinib, and binimetinib respectively. Immune checkpoint blockade inhibits the negative regulation of T cell activation, thereby unleashing antitumor T–cell responses. CTLA4, cytotoxic T–lymphocyte antigen 4; PD1, programmed cell death protein 1; PDL–1, programmed death ligand 1; RTK, receptor tyrosine kinase.

Genetic alterations affecting the RAS-RAF-MEK-ERK (Mitogen-Activated Protein Kinase, MAPK) pathway occur in approximately 40% of all human cancers. Mutations in the proto-oncogene *BRAF* and *RAS* family genes (*KRAS* and *NRAS*) are quite frequent in melanoma, CRC, anaplastic thyroid cancer (ATC) and LAC, whilst alterations affecting genes encoding MEK and ERK have rarely been identified (131, 132). For these reasons, targeting of the aberrantly activated MAPK pathway is one of the most explored therapeutic approaches in cancer. Among different neoplasms, melanoma mostly benefited from MAPK-targeted therapy. However, despite the survival advantages observed with BRAF-targeted drugs versus chemotherapy, many melanoma patients progressed within 6-7 months (133, 134), mainly due to ERK re-activation (135). Based on clinical evidence from large clinical trials (136–138), the current therapeutic strategy combines BRAF and MEK inhibition, including three FDA approved combinations for the treatment of metastatic BRAF-mut melanoma: dabrafenib plus trametinib, vemurafenib plus cobimetinib, and encorafenib plus binimetinib (**Figure 5**). Moreover, dabrafenib plus trametinib combination has been approved for the treatment of metastatic *BRAF*-mutated NSCLC and metastatic/unresectable *BRAF*-mutated ATC.

More recently, immunotherapies with checkpoint blockade Abs directed against PD-1 and cytotoxic T-lymphocyte-associated antigen 4 (CTLA-4) have revolutionized the treatment of patients with metastatic cancer including melanoma (139) (**Figure 5**). Even though the optimal sequence of targeted therapy and immunotherapy for the treatment of patients with *BRAF*-mutated melanoma is still under investigation in clinical trials [DREAMseq (NCT02224781) and SECOMBIT (NCT02631447)], currently the American Society of Clinical Oncology and the European Society of Medical Oncology guidelines recommend both therapies as first-line treatment for metastatic melanoma (140, 141). Due to the lack of mechanistic knowledge indicating the best first-line therapy to adopt, many centers treat these patients with targeted therapy first, and then switch them to immunotherapy on progression. However, patients who relapse on MAPK inhibition show a lower overall response rate (ORR) to immunotherapy compared with MAPKi naïve patients (142–144). In line with this, melanomas with acquired resistance to MAPK inhibitors show CD8 T-cell deficiency/exhaustion and loss of antigen presentation functions, which suggests cross-resistance to anti-PD1/Programmed Death-Ligand 1 (PD-L1) immunotherapy (145–147). More recently, a cancer cell-instructed, immunosuppressive tumor microenvironment lacking functional CD103^+^ dendritic cells that preclude an effective T cell response has been described in melanoma patients and mouse models (148). This mechanism is involved in the cross-resistance between MAPK inhibitors and subsequent immunotherapies (**Tables 4**, **5**). Mechanistically, patients displaying MAPK re-activation who progress on dual BRAF/MEKi, also exhibits an enhanced transcriptional output driving immune evasion.

**Table 5:** Cross–resistance in MAPK–targeted therapies.

Previous agent	Subsequent agent	Ref.	Type of study	Proposed mechanism	Supporting literature
Dabrafenib Dabrafenib+trametinib	Anti–PD1 Anti–CTLA4	*Haas L*, *Nat Cancer 2021* (148)	RAFi and RAFi/MEKi resistant melanoma mouse model + Patient analysis	Reprogramming of MAPK transcriptional output driving immunosuppressive microenvironment that lacks functional CD103^+^ dendritic cells	*Ackerman A, Cancer 2014* (142) *Johnson DB, J. Immunother 2017* (143) *Tétu P, Eur J Cancer 2018* (144) *Mason R, Pigment Cell Melanoma Res 2019* (145) *Hugo W, Cell 2015* (146) *Pieper N, Oncoimmunology 2018* (147)
Vemurafenib	Dacarbazine	*Erdmann S*, *Sci Rep 2019* (149)	Patient–derived resistant melanoma cell model	Reactivation of MAPK pathway and enhanced activation of PI3K/AKT signalling	
Dabrafenib Vemurafenib	Radiotherapy	*Shannan B*, *Eur J Cancer 2019* (151)	Patient–derived melanoma cell model + Observational	Enrichment of H3K4 demethylase JAR–ID1B/KDM5B, that regulates the transcription of genes favoring cell survival	

Another noteworthy cross-resistance event between unrelated drugs that deserves mention, has been reported between the BRAF inhibitor vemurafenib and dacarbazine chemotherapeutic in a patient-derived *BRAF*-mutated melanoma cell model (149) (**Tables 4**, **5**). In this case, dacarbazine-resistant cells re-activate the MAPK pathway by autocrine IL-8 cytokine, thereby sustaining cross-resistance to vemurafenib. By contrast, desensitization of vemurafenib-resistant cells to dacarbazine is mediated by enhanced AKT serine/threonine kinase signaling.

Brain metastases affect approximately 50% of stage IV melanoma patients requiring the combination of MAPK inhibition or immunotherapy with radiotherapy protocols (150). Cross-resistance between combined MAPK inhibition and radiotherapy has also been observed (**Tables 4**, **5**), but the extent may vary depending on the treatment sequence (151). Shannan and colleagues reported a higher rate of tumor relapse in preclinical cell models that were first treated with BRAF inhibition followed by radiotherapy compared to the reverse sequence. At the molecular level, the histone H3K4 demethylase JARID1B/KDM5B is more frequently upregulated following BRAF inhibition and predicts cross-resistance towards radiotherapy.

## Real-World Data as a Tool to Identify Cross-Resistance

It is increasingly evident that, due to the recent rapid drug development, pivotal clinical trials might not have explored the full spectrum of the cancer population. A significant proportion of cancer patients cannot be enrolled in clinical trials due to stringent exclusion criteria, even though they are still treated in clinical practice (152). Conversely, patients enrolled in clinical trials exploring (for instance) a second-line treatment could not have necessarily received current first-line treatments. Consequently, there is an unmet medical need for additional clinical practice information when choosing the optimal sequence of new anticancer agents. RWD can potentially address this knowledge gap by providing a good deal of information concerning specific drug scheduling.

RWD is referred to data collected from sources outside of conventional research settings, including electronic health records, administrative claims, tumor registries, daily clinical routine (153), and information related to disease status, treatments and their sequence, safety, concomitant medications, comorbidities, or to cancer patient population not extensively enclosed in randomized clinical trials (RCTs). As such, RWD is gaining increasing interest for the potential to provide additional evidence that can complement and support the data from RCTs.

RWD has significantly contributed to highlighting many cross-resistance events. These include the reduction in T-DM1 activity observed in BC patients previously treated with dual HER2 blockade by pertuzumab plus trastuzumab, as discussed in detail above. Not only did observational studies in the real-world setting (47–51) highlight cross-resistance but they also revealed another critical issue: due to concomitant approval of pertuzumab and T-DM1, none of the patients enrolled in the EMILIA and Th3resa trials (T-DM1 registrative studies) had previously received pertuzumab. Consequently, at the time of T-DM1 approval, clinical data on its efficacy in pertuzumab-pretreated patients was lacking.

Cross-resistance among ARSIs and between ARSIs and taxane-based chemotherapy in PC have been extensively addressed in this review. Recently, a systematic review (154) has explored optimal treatment sequencing of abiraterone acetate and enzalutamide in chemotherapy-naïve mCRPC patients. The analysis was conducted with RWD from 17 observational studies and showed a favorable trend in outcomes and cost effectiveness for the sequence abiraterone acetate-enzalutamide compared to enzalutamide-abiraterone acetate.

In addition, RWD contributed in highlighting cross-resistance between ARSIs enzalutamide and abiraterone acetate (155) and suggested a reduced efficacy of sequential ARSI treatment in chemotherapy pretreated patients.

Another relevant contribution deriving from RWD was the demonstration of lower efficacy of immunotherapy in BRAF-mutated metastatic melanoma patients relapsing on MAPK inhibition compared with MAPKi naïve patients (144).

Finally, the real-world experience in *EGFR*-mutated NSCLC demonstrated that sequential afatinib and osimertinib was beneficial in prolonging the chemotherapy-free interval in patients with T790M acquired resistance (156).

One challenging and unresolved issue relates to patients affected by advanced hepatocarcinoma (HCC). The first-line treatment of these patients is represented by the combination of anti-VEGF bevacizumb and anti-PD-L1 atezolizumab mAbs, that showed significant OS benefit over the multikinase inhibitor (MKI) sorafenib previously used in this setting, which was thereby approved by the FDA in 2020 (157). Data on efficacy and safety were subsequently confirmed by RWD analyses (158). Many therapeutic options are available for the second-line treatment of these patients, including a MKI, mAbs, or anti-PD1 agents such as cabozantinib, ramucirumab, nivolumab with or without the anti-CTLA4 ipilimumab (159). At present, the optimal second-line treatment has not yet been defined. An observational retrospective study reported comparable efficacy of second-line sorafenib and lenvatinib (160). Data from the real-world setting could help define the optimal sequence of treatments.

## Strategies to Overcome Cross-Resistance

### Molecular Re-Evaluation of Recurrences as a Strategy to Refine Clinical Trials Design

The loss of target on HER2-targeted therapy is a widely recognized issue that has been discussed above for both BC (36–47) and GC (60–62). Provided that the knowledge of the underlying mechanisms is of paramount relevance, this evidence offers the opportunity to reconsider the strategies behind the design of RCTs. Indeed, many of these studies investigate the efficacy of new therapeutic approaches in metastatic/recurring patients stratified based on the molecular features of primary tumors. Interpreting the results generated from these trials could lead to sub-optimal clinical decision-making.

An emblematic example of this is the failure of the randomized phase II study WJOG7112G (T-ACT). The aim of the study was to explore the efficacy of paclitaxel with or without trastuzumab in 99 patients with HER2+ advanced GC who had disease progression after first-line chemotherapy with trastuzumab. Median PFS and OS were not significantly different between the two groups. In this case, loss of HER2 has been reported as a possible explanation for failure. Indeed, when HER2 status was re-evaluated in tumor biopsy specimens from 16 patients following disease progression, HER2 loss was observed in 11 patients (69%) (161).

In the specific case of HER2-targeted therapy, re-evaluating the HER2 status at the time of disease progression would be required (43). Supporting this, an ongoing phase II, open-label, single arm trial aimed at evaluating the efficacy and safety of T-Dxd in Western GC patients progressed with a trastuzumab-containing regimen (DESTINY-Gastric02, NCT04014075) required patients to be re-tested for HER2 positivity before being treated with T-Dxd.

The Darwinian selection hypothesis assumes that cancer therapy selects pre-existing mutant cells that overtake the bulk cell population. However, this is a simplified mechanism that does not account for therapy resistance alone. In a more complicated scenario, genetic alterations and changes in the gene expression state often emerge under the selective pressure exerted by the therapy itself, fueling the increasingly aggressive behavior of recurring tumors (162).

On this basis, a molecular re-evaluation of patient recurrences is of paramount importance in order to identify subsets of patients to be included in RCTs where unfortunately re-biopsy is not feasible in most cases. Recently, minimally invasive liquid biopsy for the selection of patients for targeted therapies has demonstrated equivalent clinical utility to that of invasive tumor tissue testing (163). The analyses of cell-free tumor DNA (ctDNA) allows a much more rapid identification of actionable mutations compared to tissue profiling. Specifically, the ctDNA analysis has been exploited to show the acquisition of specific mutations on emerging resistance to targeted therapy (164). Currently, a few FDA diagnostic tests have been developed to provide tumor mutation profiling on NSCLC, BC, and ovarian cancer. These tests have been used to select patients for targeted therapy in the advanced setting. At the moment, global efforts aim to obtain standardized procedures for liquid biopsy tests in order to allow their rapid implementation into clinical practice. The future use of this high-potential tool will rapidly help match patients for clinical trials as well as for proper clinical decision-making.

### Identification of Collateral Sensitivities

The emergence of evolutionary dynamics (165, 166) and nongenetic reprogramming of TME (167) in therapy resistance provide a field of action for possible subsequent therapy. Interestingly, available pre-clinical and clinical evidence indicate cases of collateral sensitivities that are novel, exploiting vulnerabilities emerging concurrently with therapy resistance.

In the current scenario where most patients are still treated with traditional chemotherapy, several cases of collateral sensitivities between chemotherapeutic agents have been reported. Pre-clinical and clinical evidence suggest that cisplatin resistance can result in sensitivity to paclitaxel, and vice-versa (168, 169). Despite the underlying mechanism remaining unknown, combining the two drugs has been proven to be effective in lung, ovarian, skin, breast, and head and neck tumors (170). Similarly, vinblastine-resistant cell lines are sensitive to paclitaxel, and vice-versa (171). In this case, the two drugs exert opposing mechanisms of action (vinblastine destabilizes microtubles while paclitaxel stabilizes microtubles); resistance can stem from stabilizing (vinblastine) or destabilizing (paclitaxel) mutations in α- and β- tubulin.

In the context of targeted therapies, the first collateral sensitivity network was provided by Dhawan and colleagues in 2017. In an attempt to characterize collateral sensitivities to several TKIs in Anaplastic Lymphome Kinase (ALK)-positive NSCLC, they found that cell lines resistant to first-line TKIs are often sensitized to the chemotherapeutic drugs etoposide and pemetrexed (172). More recently, the same authors showed that resistance to chemotherapy in Ewing’s sarcoma cell lines is associated with sensitivity to the histone demethylase 1 inhibitor SP-2509 (173).

These findings have fueled further exploration in pre-clinical models, consequently expanding our knowledge in this field. Melanoma cells which developed resistance to MAPKi showed enhanced susceptibility to platinum-based drugs such as cisplatin and carboplatin, that is inversely correlated with the expression level of the p53 family member TAp73. Mechanistically, low TAp73 expression level results in reduced efficacy of the nuclear excision repair system and enhanced sensitivity towards platinum-based cytostatic agents (174). Similarly, resistance to BRAF/MEK inhibitors is associated with increased levels of reactive oxygen species and enhanced efficacy of the histone deacetylase (HDAC) inhibitor vorinostat in resistant cell and mouse models, as well as in patients (175). Accordingly, a pilot study in patients demonstrated that treating BRAF inhibitor-resistant melanoma patients with HDAC inhibitors killed the drug-resistant cell population (175).

In EGFR-mutant LUAD cells, acquired resistance in response to EGFR inhibitors requires Aurora Kinase A activity, and is therefore associated with increased sensitivity to Aurora kinase inhibitors (176).

In the context of BC, HER2 mutations, resulting in cross-resistance between HER2-targeted therapies, are associated with higher efficacy of some irreversible HER2 TKIs such as neratinib and pyrotinib both in HER2-amplified (65, 66) and HER2 non-amplified (177, 178) BC. In a panel of 115 cancer cell lines, neratinib was the most effective against HER2-mutant cell lines among HER2-targeted TKIs (179). The phase II SUMMIT trial concluded that neratinib in combination with fulvestrant is clinically active in heavily pretreated HER2-mutant HR+ BC patients (180). Thus, HER2 mutations might be predictor of benefit from Neratinib TKi. By employing a cell-model and 3D *ex vivo* organotypic culture model, Singh and colleagues showed that a high level of the detoxifying enzyme Sulfotransferase Family 1A Member 1 (SULT1A1) confers resistance to Tamoxifen and collateral sensitivity to the anticancer compounds with SULT1A1-dependent activity RITA (Reactivation of p53 and Induction of Tumor Cell Apoptosis), aminoflavone (AF), and oncrasin-1 (ONC-1) (181).

In pancreatic ductal adenocarcinoma (PDAC) patient-derived organoids, chemotherapy-induced vulnerabilities were investigated that highlighted increased sensitivity to MEK inhibition, driven by tumor plasticity in response to chemotherapy regimen FOLFIRINOX (combination therapy with Folinic Acid, fluorouracil, irinotecan, and oxaliplatin) (182). In this case, therapeutic vulnerabilities were identified by unbiased drug screening experiments and did not seem to be associated with a specific genetic marker. This is a significant indication that molecular deregulations alone may not account for collateral sensitivities, and an additional functional layer is needed for precision oncology. Similarly, some of these studies suggested the involvement of rapidly changing gene expression regulations in the response to drugs rather than providing specific mechanisms for collateral sensitivities.

On the other hand, it is worth considering that our current knowledge of potentially therapeutically targetable dependencies is still limited and recurrently mutated genes account for this burden only partially (183). New emerging categories of cancer targets that include cell-autonomous and tumor microenvironment (TME)-mediated targets, are likely to result in the development of novel targeted agents and thereby novel therapeutic options in the near future.

In this scenario, identifying predictive biomarkers to stratify patients who would likely benefit from cancer therapies is currently an active field of investigation. In this regard, it is expected that many categories of drug-induced deregulation may be considered, spanning from genetic/epigenetic deregulations to nonmutational, functional alterations.

### Investigation of Rational Mechanistic-Based Cancer Treatment Regimens

One strategy used to overcome resistance to targeted therapies is represented by combination therapy simultaneously blocking parallel or alternative pathways activated in cancer cells. However, due to the complexity of signaling networks, efficient screening for effective targeted combination therapies is a challenging issue, which is further complicated by the need to address clinically relevant doses and dosing schedules that can impact the emergence and evolution of resistance.

Mathematical modeling represents a reasonable tool for testing clinically relevant drug combinations prior to investment in clinical trials. Branching process models had been used to study resistance to chemotherapy in tumor cell populations as early as in the 1980s (184). Since then, other groups exploited mathematical modeling to characterize drug resistance and investigate potential effective schedules in order to minimize the development of acquired resistance (185, 186). More recently, a computational modeling platform and software package have been developed for identifying optimum dosing for combination treatments of oncogene-driven cancers (187).

In addition, refining doses and scheduling in combination therapy is of paramount importance in order to reduce the emergence of resistance and cross-resistance. Currently, some rational combination strategies are under investigation which have the potential to reach this goal, thereby improving cancer therapy.

One of these strategies is represented by multiple low-dose treatment. So far, the vast majority of novel cancer drugs are developed as single agent therapies and are delivered to patients at a maximum tolerated dose. In case of drug combinations, it is generally believed that each drug should be used according to the same criteria. However, recent available evidence indicates that multiple low-dose treatment can be effective: in EGFR-mutant lung cancer, vertical targeting of EGFR signaling pathway with three or four drugs can be effective even when the drugs are used at 20% of the single agent concentration (188). Similarly, dual RAF/ERK low-dose was effective in KRAS-mutant cancers (189). In the specific case of vertical targeting of multiple nodes of a signaling pathway, the adoption of a low-dose regimen reduces the selective pressure on these nodes and the eventual emergence of resistance mutation.

Sequential drug treatment is conceptually based on the induction of a major vulnerability by the first drug, that is targeted by a second drug to kill tumor cells. According to this principle, sequential, but not simultaneous, treatment of triple-negative BC cells with EGFR inhibitors and DNA-damaging drugs results in efficient cell killing (190). In metastatic BC patients, pretreatment with cisplatin and doxorubicin resulted in enhanced responses to anti PD-1 therapy (191). Also, sequential drug treatment for combination immunotherapies is supported by preclinical data (192).

Parallel to studies of drug scheduling, drug holidays, or metronomic therapy, has also been proposed as a strategy to limit the development of resistance in cancer treatment (193, 194). It is conceptually based on the principle that upon removal of therapy, cancer cells do not need to develop advantageous adaptations that drive resistance. From a molecular point of view, this effect can be achieved by reversible adaptation (194) or mutation–independent phenotypical variations (195). In preclinical models of melanoma, intermittent dosing with BRAF inhibitors results in delayed emergence of resistance as compared to continuous dosing (196). However, conflicting results derived from clinical data indicating that intermittent dosing is inferior to continuous administration, highlighted that careful attention must be paid when translating dosing and treatment schedules from preclinical models to humans (197).

Overall, these efforts are intended to lay a solid mechanistic basis for drug combination regimens and avoid clinical trials investigating combination treatments without a rational basis.

## Concluding Remarks

The emergence of drug resistance has proven to be a major obstacle from the first available cancer chemotherapies available right up to the latest, rapidly developing targeted therapies. Next–generation sequencing and computational data analysis approaches have revealed that genomic instability sustains tumor heterogeneity which allows human cancers to escape from therapies and develop resistance. An increasing number of therapeutic possibilities available entails further levels of complexity and cross–resistance to secondary or subsequent therapies can occur, impacting on patient outcomes and survival rates.

The emergence of cross–resistance among drugs acting on a shared target may occur. In response to the first specific agent, threatened cancer cells acquire deregulation or mutation to the target guaranteeing not only escape from therapy, but also cross–resistance to a secondary drug acting on the same target. The reversible/irreversible nature of target deregulation deserves further investigation. It has been reported that the time interval between consecutive HER2–targeted therapies in BC may play a key role in cross–resistance, as HER2 downregulation is associated with a shorter interval between the last HER2–targeted agent administered and the time of HER2 assessment (45). At the moment, we do not know whether a reversible loss of HER2 may be induced by HER2–targeting agents and to what extent the reversible (internalization/nuclear translocation) and irreversible (clonal selection) loss of HER2 could impact the efficacy of subsequent therapy.

Many cross–resistance events have been reported between therapies that exert different modes of action. Highly representative of current practice is the recent cross–resistance reported between dual BRAF/MEK inhibition and subsequent immunotherapies. One fundamental point with this finding is the acquisition of cross–resistance during MAPKi treatment, questioning once again the hypothesis of clonal selection of resistant cells pre–existing before therapy.

It is critical to decipher the underlying mechanism(s) of cross–resistance in order to overcome it. To this aim, a powerful tool is represented by recent studies that exploit complex preclinical cell models including not only primary tumor cells, but also cells from fibroblastic, vascular, and immune compartments. These models resemble the tumor heterogeneity and the contribution of TME and immune compartments to cross–resistance dynamics which are typically observed *in vivo* (198, 199) and therefore represent an ideal tool for investigating new vulnerabilities.

Accordingly, the conceptual design behind RCTs needs to swiftly and adequately incorporate the growing knowledge of cancer evolution in response to therapy. Experience from past RCTs indicates an urgent need to reconsider the molecular landscape of recurring tumors and exploit newly acquired targetable vulnerabilities for making more effective therapeutic decisions.

## Author contributions

GB, RL, PV, and FL were involved in manuscript drafting. GB, SS, and MM–S revised the manuscript. All authors contributed to the article and approved the submitted version.

## Funding

This work was supported by Funds Ricerca Corrente 2022 from Italian Ministry of Health.

## Conflict of interest

The authors declare that the research was conducted in the absence of any commercial or financial relationships that could be construed as a potential conflict of interest.

## Publisher’s Note

All claims expressed in this article are solely those of the authors and do not necessarily represent those of their affiliated organizations, or those of the publisher, the editors and the reviewers. Any product that may be evaluated in this article, or claim that may be made by its manufacturer, is not guaranteed or endorsed by the publisher.
