# Corrigendum: Cross-resistance among sequential cancer therapeutics: An emerging issue

**DOI:** 10.3389/fonc.2022.1063651

**Published:** 2022-10-31

**Authors:** Rossella Loria, Patrizia Vici, Francesca Sofia Di Lisa, Silvia Soddu, Marcello Maugeri-Saccà, Giulia Bon

**Affiliations:** ^1^ Cellular Network and Molecular Therapeutic Target Unit, IRCCS Regina Elena National Cancer Institute, Rome, Italy; ^2^ Unit of Phase IV Trials, IRCCS Regina Elena National Cancer Institute, Rome, Italy; ^3^ Medical Oncology A, Department of Radiological, Oncological, and Anatomo-Pathological Sciences, Umberto I University Hospital, University Sapienza, Rome, Italy; ^4^ Division of Medical Oncology 2, IRCCS Regina Elena National Cancer Institute, Rome, Italy

**Keywords:** targeted-therapy, cancer therapeutics resistance, cross-resistance, sequential therapeutics, personalized oncology

In the published article, there was an error in affiliations 1, 2, and 4. In these affiliations, “Istituto di Ricerca e Cura a Carattere Scientifico Regina Elena National Cancer Institute, Rome, Italy” has been changed to “IRCCS Regina Elena National Cancer Institute, Rome, Italy”.

The authors apologize for this error and state that this does not change the scientific conclusions of the article in any way. The original article has been updated.

## Publisher’s note

All claims expressed in this article are solely those of the authors and do not necessarily represent those of their affiliated organizations, or those of the publisher, the editors and the reviewers. Any product that may be evaluated in this article, or claim that may be made by its manufacturer, is not guaranteed or endorsed by the publisher.

